# Pheromonal Cues Deposited by Mated Females Convey Social Information about Egg-Laying Sites in *Drosophila Melanogaster*

**DOI:** 10.1007/s10886-016-0681-3

**Published:** 2016-03-19

**Authors:** Claire Duménil, David Woud, Francesco Pinto, Jeroen T. Alkema, Ilse Jansen, Anne M. Van Der Geest, Sanne Roessingh, Jean-Christophe Billeter

**Affiliations:** Groningen Institute for Evolutionary Life Sciences, University of Groningen, 9700CC Groningen, The Netherlands

**Keywords:** *Drosophila melanogaster*, Communication, Social learning, Oviposition, Pheromones

## Abstract

Individuals can make choices based on information learned from others, a phenomenon called social learning. How observers differentiate between which individual they should or should not learn from is, however, poorly understood. Here, we showed that *Drosophila melanogaster* females can influence the choice of egg-laying site of other females through pheromonal marking. Mated females mark territories of high quality food by ejecting surplus male sperm containing the aggregation pheromone *cis*-11-vaccenyl acetate (cVA) and, in addition, deposit several sex- and species-specific cuticular hydrocarbon (CHC) pheromones. These pheromonal cues affect the choices of other females, which respond by preferentially laying eggs on the marked food. This system benefits both senders and responders, as communal egg laying increases offspring survival. Virgin females, however, do not elicit a change in the egg-laying decision of mated females, even when food has been supplemented with ejected sperm from mated females, thus indicating the necessity for additional cues. Genetic ablation of either a female’s CHC pheromones or those of their mate results in loss of ability of mated females to attract other females. We conclude that mated females use a pheromonal marking system, comprising cVA acquired from male ejaculate with sex- and species-specific CHCs produced by both mates, to indicate egg-laying sites. This system ensures information reliability because mated, but not virgin, females have both the ability to generate the pheromone blend that attracts other flies to those sites and a direct interest in egg-laying site quality.

**Electronic supplementary material:**

The online version of this article (doi:10.1007/s10886-016-0681-3) contains supplementary material, which is available to authorized users.

## Introduction

Selecting an appropriate egg-laying site is crucial to oviparous females, as the quality of a site will determine their offspring’s chance of survival. Females may select a suitable site through trial-and-error or imitate the choice of other individuals, a strategy called social learning (Bandura 1971). A problem with this social transfer of information is that other individuals are not always reliable, since they might be wrong or even deceitful (Laland 2004). In order to choose between individual and social learning, it is, therefore, essential to recognize if information coming from others is trustworthy.

The selection of egg-laying sites by *Drosophila melanogaster* females is a good behavior for studying the basic mechanisms of social learning. Although female fruit flies use physical environmental cues to select individual egg-laying sites (Gou et al. 2014; Joseph et al. 2009; Laturney and Billeter 2014; Yang et al. 2008), they also can use social information. Females can choose egg-laying sites by observing a demonstrator female laying eggs on a food patch (Sarin and Dukas 2009), through direct social interaction with an experienced female that communicates which site to choose (Battesti et al. 2012), or through chemical cues left behind by sender flies that function as a public source of information for responders (Lin et al. 2015; Wertheim et al. 2002a). The mechanisms underlying identification of reliable senders and the means by which they communicate favorable egg-laying sites are, however, poorly understood.

In this work, we investigated the mechanisms underlying the production, by senders, and sensing, by responders, of cues used as public information about egg-laying sites. This social exchange of information in *Drosophila* appears to be a form of mutualism in that both senders and responders benefit, as communal egg laying increases offspring survival through cooperation between larvae in fending off fungal growth on food and better resource exploitation (Wertheim et al. 2002b). These benefits are, however, density-dependent, as communal egg laying is detrimental to population growth under conditions of overcrowding and resource over-exploitation (Etienne et al. 2002; Wertheim et al. 2002b). For this reason, we specifically studied the mechanism underlying social learning *via* public information under low density conditions, in which communal egg-laying is beneficial (Wertheim et al. 2002a). Because fruit flies aggregate on food through an aggregation pheromone, *cis*-11-vaccenyl acetate (cVA), found in males and mated females (Bartelt et al. 1985; Wertheim et al. 2002b), we explored how flies communicate about communal egg-laying sites through chemical cues.

## Methods and Materials

### Drosophila Stocks and Genetics

Flies were reared on medium containing agar (10 g/l), glucose (167 mM), sucrose (44 mM), dried yeast (35 g/L), cornmeal (15 g/L), wheat germ (10 g/L), soya flour (10 g/L), molasses (30 g/L), propionic acid (5 mM), and Tegosept (2 g/L). This medium is called “fly food” in this report. Flies were raised in a 12:12 h L/D cycle (LD 12:12) at 25 °C. Females were collected, using CO_2_ anaesthesia, within 3 hr of eclosion to ensure virgin status, and aged in same-sex groups of 20 in vials, with food for 6 d prior to testing.

We used the wild-type strain *Oregon*-*R* for all experiments, as well as the following transgenic and mutant strains: oenocyte-ablated (oe^−^) flies (p{*PromE*(*800*)-*gal4*},*p*{*TubP*-*gal80*^*ts*^}/*p*{*UAS*-*hid*},*p*{*UAS*-*nuclearGFP*}) and control flies (p{*PromE*(*800*)-*gal4*},*p*{*TubP*-*gal80*^*ts*^}/*p*{*UAS*-*nuclearGFP*})(Billeter et al. 2009). *Ir8a*^−^ null mutant (*w*^−^,*Ir8a*^*1*^), *Ir8a* rescue (*w*^−^,*Ir8a*^*1*^,*p*{*Ir8a*^+^}) (Abuin et al. 2011), *Orco*^−^ null mutants (*w*^−^;*Orco*^*1*^), and *Orco*^−^ rescue (*w*^−^;*Orco*^*1*^,*pBac*{*Orco*^+^}) (Silbering et al. 2011) were gifts from R. Benton. *w*^−^; *p*{*ProtamineB*-*eGFP*} (Jayaramaiah Raja and Renkawitz-Pohl 2005) was obtained from the Bloomington stock centre.

### Social Transmission of Information about Egg Laying Site Assay

**Fig. 1:**
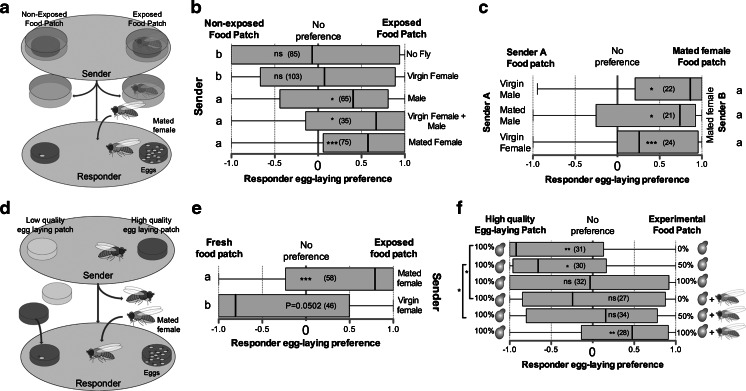
Mated female *Drosophila melanogaster* transfer information about favorable egg-laying patches. **a** Bioassay for testing chemical cues about egg-laying sites. One of two food patches of identical nutritional quality is exposed to a single sender fly, which is discarded after 6 hr, and a mated female is introduced in the arena and allowed to lay eggs on both patches. **b** Egg laying preference of a responder female to two food patches, one of which was previously exposed to flies of the indicated sex and mating status. **c** Egg-laying preference of a responder female to two food patches of identical nutritional quality; as for (*b*) except that both patches have been previously exposed to flies of the indicated sex and mating status. **d** Bioassay of the ability of senders to transfer information about favorable egg-laying sites. Similar to the bioassay in (*a*), except that the sender female is free to explore both food patches and the two food patches are of different quality (yeast added to the high quality patch). **e** Egg-laying preference of a mated female to two high quality egg-laying patches, one of which was previously housed with either a mated or a virgin female free to visit both patches. **f** Egg-laying preference of a mated female to one high quality egg-laying food patch containing 100 % yeast (left side of the graph), and a second food patch containing one of three possible concentrations of yeast and that had been or not previously exposed to a mated female (right side of the graph). Errors bars indicate minimum and maximum data points. Number of replicates is indicated in brackets. Preference for a site is indicated in each box plot by asterisks, as determined by a two-tailed exact Wilcoxon signed rank test (*n.s*. = not significant; ***, *P* < 0.001). Different letters (*a*, *b*) on the side of the box plots indicate differences between groups, as determined by a logistic regression model; see Table S4 for full statistics

Females and males used as senders were mated at 23 °C at the onset of the light phase [Zeitgeber Time (ZT) 0 or 9:00 in our case] in mating arenas, containing groups of 6 virgin females and 6 virgin males, consisting of a disposable Petri dish (55x8 mm) containing a circular fly food patch (25x1mm). To ensure that the social experience of senders prior to testing was as similar as possible, male and virgin female senders were single-sex housed in mating arenas prior to experiments. Single senders were transferred 2 hr later, at ZT2, to a 90x12 mm Petri dish containing two food patches (25x1mm) placed 40 mm apart. Food patches were made according to the fly food recipe. All food patches contained all fly food ingredients except for yeast, which was sometimes removed or added at different concentrations, as indicated in the text. Single senders were either allowed to walk freely or were forced to occupy one food patch by placing them under the cover of a 35x10 mm Petri dish for 6 hr after which flies and covers were discarded (Fig. 1a). Dishes in which females laid eggs were discarded. A naïve mated responder female was transferred at ZT 9 to the dish previously visited by a sender and allowed to walk freely in the dish and to lay eggs for 20 hr (Fig. 1a). The number of eggs on each food patch was counted at the end of the experiment and patch preference was determined using the following formula:

$$ \frac{\left(\#\  eggs\  on\  the\  marked\  food\  patch-\#\  eggs\  on\  the\  unmarked\  food\  patch\right)}{Total\ \#\  eggs} $$

### Fly Tracking

To monitor the location of flies in relation to the food patches, photographs of the 90 mm assay dish containing two food patches were taken at 2-min intervals using Logitech 910C webcams controlled by the SecurityMonitor Pro software (Deskshare Inc). Sender flies were recorded for 6 hr and responders for 12 hr. Fly coordinates in each time-lapse photo were manually determined using the software ImageJ 1.48v (Schneider et al. 2012). Using these coordinates, the distance of each fly to both food patches was determined at each time point by trigonometry. For Patch preference assays, flies positioned <27 mm from the center of the food patch previously marked or exposed to a sender fly were considered “near the food patch”, the others as “away from food”, and a patch preference was calculated using the following formula:

$$ \frac{\left(\#\  position s\  near\  food-\#\  position\  away\  from\  food\right)}{Total\ \#\  position s} $$

### Ejected Sperm Observation

The location of sperm ejection was monitored using a MZ10F stereomicroscope equipped with filters for UV light and Green Fluorescent Protein (GFP) detection (Leica Microsystems Ltd; Germany). Images were taken using a Leica DC250 camera and the Leica Application suite software.

### Chemical Extraction and Analysis

For hydrocarbon analysis, single flies were placed in glass microvials containing 50 μl of an internal standard solution, made by dissolving 10 ng/ml of octadecane (C_18_) and 10 ng/ml of hexacosane (C_26_), in hexane. Ejected sperm were collected using a clean metal pin and transferred to a glass microvial as above. Vials were vortexed at minimum speed for 2 min, and the fly, but not ejected sperm, was removed using a clean metal pin.

To analyse CHCs deposited in glass dishes, 4 *Oregon*-*R* mated or virgin females were housed together in a 40x10 mm glass Petri dish for 4 hr. The glassware was rinsed with 200 μl hexane and poured into a 200 μl glass microvial. Each extract was reduced to 40 μl under nitrogen flow and 40 μl of internal standard added.

The resulting hydrocarbon extracts were analyzed using an Agilent 7890 gas chromatograph (GC) with a flame ionization detector, an Agilent DB-1 column (diam.: 0.180 mm; film 0.18 μm), and a splitless injector set at 250 °C with helium as carrier gas (flow: 37.2 cm.sec^−1^). The column oven temperature was programmed from 50 °C, for 1.5 min, to 150 °C, at 10 °C.min^-!^, then to 280 °C at 4 °C.min^-!^, and held for 5 min. ChemStation software (Agilent technologies) was used to integrate compounds based on peak areas relative to the internal standard C_26_, as described in Krupp et al. (2008).

### Pheromonal Bioassay

Mated females were extracted as described above, except in 10 μl hexane and concentrated by nitrogen flow to 2 μl, which was then deposited on a cellulose chromatography paper (Ch1, Whatman) circle (diam.: 5 mm) and air dried for 30 min to evaporate the hexane. The impregnated circle was deposited on one of the food patches, while a circle impregnated with hexane only was deposited on top of the second food patch. Cuticular hydrocarbon transfer was determined by extracting impregnated paper circles, as described above, and quantifying by GC.

### Statistical Analysis

Graphs and statistical analyses were performed using GraphPad Prism 5 (GraphPad Software Inc., USA) and R 3.2.0 (The R Foundation for Statistical Computing). Statistics were performed in two stages. The first stage tested preference data for each group for whether flies chose one food patch over the other. Preference indices were tested against the null hypothesis that responders did not make a choice, using an exact two-tailed Wilcoxon Signed Rank tests, with no preference set at “0”. A significant effect indicated a preference for, or stronger association with, one food patch over the other. The second stage compared groups within each experiment. Because our experiments generated choice data, a quasibinomial logistic regression was applied on the proportion of eggs/positions, near each food patch. To be able to apply a binomial distribution, data were arranged as a matrix of 2 vectors: numbers of success (number of eggs on the marked food patch), and numbers of failure (number of eggs on the other food patch). For patch occupation preference, the number of positions was assembled as for eggs. The explanatory variable included the type of marking applied on one of the food patches (*i.e*., sender, sperm ejection, female extract), food quality or the type of responder used, and their interaction whenever relevant. *Post*-*hoc* testing of between-group differences was performed by comparing a control group (see figure legends) to each of the other groups. Significant predictors are summarized in the figures and reported in detail in Supplementary Table S4.

## Results

### Naïve Females Learn about Egg–laying Sites through Cues Left by Mated Females

In order to study social transfer of information about egg-laying sites, we developed an assay in which a “Sender” fly was forced to occupy one of two egg-laying patches of identical nutritional quality placed in the same dish (Fig. 1a). The sender was removed after 6 hr, a time interval in which freshly mated females do not generally lay eggs, the presence of which would be an obvious cue. A naïve “responder” mated female was housed in the dish for 20 hr, during which time she was free to move and lay eggs on both patches in absence of direct interaction with the sender. We first tested whether a patch previously exposed to a sender was preferred for egg laying, over an unexposed patch, by a responder female. Indeed, responders preferred to lay eggs on patches previously occupied by either a male or a mated female, showing that both types can act as senders (Fig. 1b). However, no preference by responders was observed to food patches previously occupied by virgin females, suggesting that not all flies are trusted senders or capable of leaving egg-laying cues (Fig. 1b). Sequentially housing a virgin female and a male on the same food patch rendered these patches as preferred egg-laying sites for responder females (Fig. 1b). This indicates that the presence of a virgin female does not block the ability of a male to affect the egg-laying preference of females.

As both males and mated females can act as senders, we next determined whether patches occupied by one of these two senders was preferred for egg laying by responder females. To show discrimination, we modified the assay to test, simultaneously, two different senders housed on food patches located opposite each other while monitoring the responder female’s egg-laying preference. Responder females preferred a food patch previously exposed to a mated female over one exposed to a male or virgin female (Fig. 1c), thus showing that mated females affect the egg-laying site choice of responder females more strongly than males. Subsequently, we focused our investigation on the mechanisms of transfer of social information between sender mated females and responder females.

Yeast is important to mated females, which feed and lay eggs on the food source, as it contains necessary nutrients for egg production and larval offspring growth (Baumberger 1917; Becher et al. 2012; Terashima and Bownes 2004). Virgin females, however, prefer food containing sugar over that containing yeast (Ribeiro and Dickson 2010). To investigate whether female senders guide responder females to food patches favorable for egg laying, we allowed a mated or virgin sender female to move freely between a food patch containing all the ingredients of our fly food minus yeast (low quality egg-laying patch) and a food patch containing all ingredients, including yeast (high quality egg-laying food patch; Fig. 1d). The low quality patch was replaced with a high quality one after removal of the sender to expose responder females to patches of identical quality for egg laying (Fig. 1d). This experiment differed from that of Fig. 1a in that the sender was free to mark one of the patches or none. Preference for the exposed patch would indicate that the sender was indicating a high-quality egg-laying patch; no preference would indicate that the sender did not mark or marked the low-quality patch. Responders preferentially laid eggs on the high quality patch exposed to a mated female (Fig. 1e), thus showing that a cue from sender mated females guides responder females to the highest quality egg-laying patch. Surprisingly, responders preferred the fresh patch over a patch exposed to a virgin female sender (Fig. 1e). The reason for this avoidance is unclear given that virgin females induced no preference in the forced-choice experiment and had no effect on cues from males (Fig. 1b). Nevertheless, these data show that mated, but not virgin, females can indicate favorable egg-laying sites.

Female senders benefit from guiding other females to lay eggs on the same food patch because aggregated oviposition enhances the survival of their offspring (Wertheim et al. 2002b). Sender females that only have access to a low quality egg-laying substrate would, therefore, benefit from guiding responder females to a low quality food patch. However, would responder females go to cues from senders on a low quality substrate, when they have access to a high quality patch? To answer this question, we modified the assay to test the egg-laying preference of responder females exposed to an unmarked high quality food patch pitted against a food patch that contained more or less yeast (0 %, 50 %, or 100 % of the yeast content of high quality food) and that had been exposed or unexposed to a sender mated female. This full factorial design showed that responder females preferentially laid eggs on unmarked food patches with high yeast content over unmarked patches with lower yeast content (Fig. 1f). Yeast concentration in the food is thus a factor determining egg-laying preference (yeast concentration: *P* > 0.001, See Table S4 for full statistics). Strikingly, housing a sender mated female on a food patch devoid of yeast rendered that food patch of equal preference to an unmarked high quality egg-laying patch (Fig. 1f). Thus, the housing of a sender mated female on a food patch had an influence on the egg-laying preference of responder females, even on low quality egg-laying patches (presence of sender mated female on food patch: *P* > 0.001, See Table S4 for full statistics), indicating that sender females will leave cues for responder females to lay eggs on low quality-egg laying patches, and that responder females will lay some of their eggs on marked low quality patches, even in the presence of a high quality egg laying patch. However, the lack of interaction between yeast concentration and presence of sender females (yeast concentration*presence of sender female: *P* = 0.7, See Table S4 for full statistics) indicates that sender females marking a low quality egg-laying patch do not reverse the preference of responder females for high quality egg-laying patch but, rather, increase the low egg-laying preference of responders for low quality egg-laying patches. Thus, the egg-laying patch decision by responders is a balance between food patch quality and the transfer of social information from sender females.

### Mated Female Preferentially Visit and Eject Sperm Next to Favorable Egg-Laying Sites

**Fig. 2:**
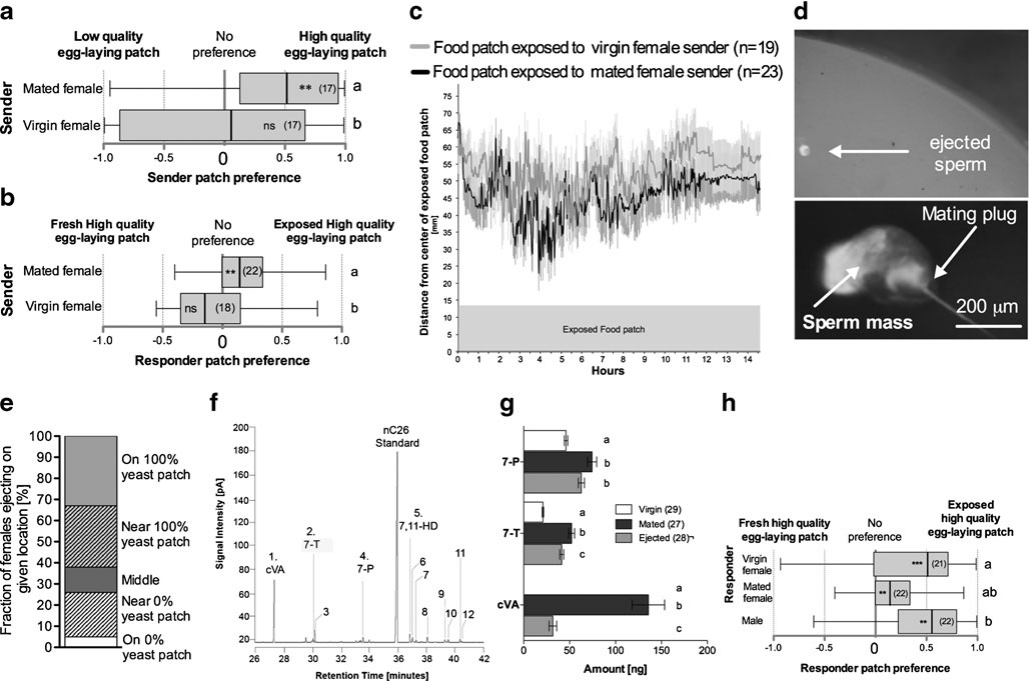
Mated females preferentially visit and eject sperm next to favorable egg-laying sites. **a** Preference of mated or virgin sender females for more time near a high (100 % yeast) or low (o% yeast) quality egg-laying patch. **b** Preference of responder mated females, in terms of location next to two egg-laying patches of equal quality, one of which was previously housed with either a mated or a virgin wild-type female. **c** Distance of responder mated females, determined every 2 min over 14 hr, next to two egg-laying patches of equal quality, one of which was previously exposed to either a mated or a virgin wild-type female. **d** Micrograph of a green fluorescent protein-fluorescing ejected sperm. The top micrograph shows an ejected sperm deposited on a patch of food, the bottom one shows the different parts of the ejected sperm. **e** Proportion of females that ejected sperm near a high (100 % yeast) or low quality (0 % yeast) egg-laying patch. **f** Gas chromatogram (flame ionization detection) of a hexane extract of a single sperm ejection. cVA = *cis*-11-vaccenyl acetate, 7,11-HD = (7*Z*,11*Z*)-heptacosadiene, 7-T = (*Z*)-7-tricosene, and 7-P = (*Z*)-7-pentacosene. Identities and quantification of the numbered peaks are given in Supplementary Table 1. **g** Mean amount of male- and female-specific cuticular hydrocarbons found in extracts of single females of the indicated mating status. Histograms labeled with different letters are different for the given compound as determined by Kruskal-Wallis ANOVA (for details, see Table S2). **h** Mated or virgin females and male responder preference between two food patches, one of which was previously exposed to a mated female. Error bars indicate S.E.M. Preference for a site is indicated in each box plot by asterisks (n.s.: not significant; ***, *P* < 0.001), as determined by a two-tailed exact Wilcoxon signed rank test. Different letters to the right of the box plots indicate differences between groups, as determined by a logistic regression; see Table S4 for full statistics

The observation that cues from mated and virgin female senders results in opposite egg-laying preferences when females are free to visit a high (100 % yeast) or low (0 % yeast) quality egg-laying patch (Fig. 1c) suggests that these senders leave different cues. To determine the nature of these cues, we tracked the time spent by female senders near the high quality patch in a dish containing high or low quality patches. Differences in frequency would indicate differential attraction or arrestment in response to these food patches. Mated females spent most of their time near high quality patches, while virgin females showed no preference (Fig. 2a). As the favored frequentation by mated female senders of the high quality patch correlated with the egg laying preference of responders (Fig. 1e), we investigated the frequentation by responder females to patches that had been freely visited by either a virgin or a mated sender female (Fig. 2b). Mated female responders spent more time near the high quality patch visited by mated female senders over the non-visited patch; by contrast mated female responders did not discriminate between the high quality patch exposed to virgin females and the unexposed patch (Fig. 2b). A correlation between the location of mated female senders and female responders, in the absence of direct interaction, suggests that mated females leave chemical cues that mark the area of the dish occupied by the high quality egg-laying patch.

Chemical cues left by senders that influence oviposition could act either by attracting responder females through oriented movement toward the food patch, or by arresting females on the food patch (Kennedy 1978). To differentiate between these two scenarios, we tracked the distance of females to the exposed food patch while responding to two high quality food patches, one unexposed and one previously exposed to either a mated or a virgin female, using the assay described in Fig. 1a. Tracking data indicated that females responding to a food patch exposed to a mated or virgin female did not stop for long periods on the food patch, indicating that sender flies do not produce cues that arrest responder females (Fig. 2c). Rather, the data suggest that a food patch exposed to a mated female sender is more attractive than a food patch visited by a virgin female, because responder females came closer to, (Fig. 2c), spent more time in (Fig. 2b), and laid more eggs (Fig. 1b) on the food patch exposed to a mated female than on the one exposed to a virgin female.

Examination under ultraviolet light of a dish containing a high and a low quality egg-laying patch after a visit by a sender mated female revealed small spots indicative of the auto fluorescence of the mating plug produced by the male ejaculate (Lung and Wolfner 2001). A mated female will eject surplus sperm and a mating plug a few hours after mating (Lee et al. 2015; Manier et al. 2010). To aid in visualizing the fate of the male ejaculate, we mated females with males that produce GFP-labeled sperm (Jayaramaiah Raja and Renkawitz-Pohl 2005). We first investigated the temporal dynamics of female sperm ejection and found that half of females ejected within 5 hr of mating (Fig. S1). This is less time than the 6 hr that senders are exposed to food patches. We then investigated the spatial distribution of ejected sperm in relation to low and high quality patches and found that >60 % of mated females ejected sperm and the mating plug (Fig. 2d) near or on the high quality patch (Fig. 2e). The male pheromone, cVA, is made in the male ejaculatory bulb and transferred from males to females during copulation (Brieger and Butterworth 1970; Butterworth 1969), and acts as an aggregation pheromone, increasing egg-laying when experimentally deposited on a food patch (Bartelt et al. 1985). Intrigued by the possibility that sperm ejection acts as a means for females to deposit cVA, we investigated the chemical content of ejected sperm. Chemical analysis of a single ejaculate revealed cVA as well as several cuticular hydrocarbons (CHCs), including the female- and species-specific sex pheromone, (7*Z*,11*Z*)-heptacosadiene (7,11-HD), and the sex pheromone components (*Z*)-7-tricosene (7-T) and (*Z*)-7-pentacosene (7-P) (Fig. 2f; Table S1 for quantification). CHCs are made independently of cVA in oenocytes, and they serve as sex- and species-specific recognition signals (Billeter et al. 2009; Billeter and Levine 2013; Brieger and Butterworth 1970; Fan et al. 2003). Analysis of the pheromonal profile of virgin females, mated females, and mated females who ejected sperm suggests that cVA is acquired through copulation and lost through ejection (Fig. 2g; Table S2), revealing the means by which mated females deposit cVA in their environment (Bartelt et al. 1985). As described in previous reports (Farine et al. 2012; Scott 1986), we observed that females also acquired an increase in their overall levels of 7-T and 7-P through mating (Fig. 2g).

The aggregation pheromone content of ejected sperm and its location next to high quality egg-laying patches suggested that sperm ejected by mated females might act as a signal to attract other flies. We monitored the location of males, as well as virgin and mated females, with respect to the location of ejected sperm in a dish containing two high-quality egg-laying patches: one that had been exposed to a free moving mated female and received an ejected sperm, and one unexposed to a mated female (Fig. 2h). Males and females, whether mated or virgin, all spent more time in the vicinity of the food patch containing the ejected sperm than the unmarked one (Fig. 2h), thus indicating that flies react to food patches on which mated females ejected sperm.

### Combinatorial Chemical Cues Guide Females to Egg-Laying Sites

**Fig. 3:**
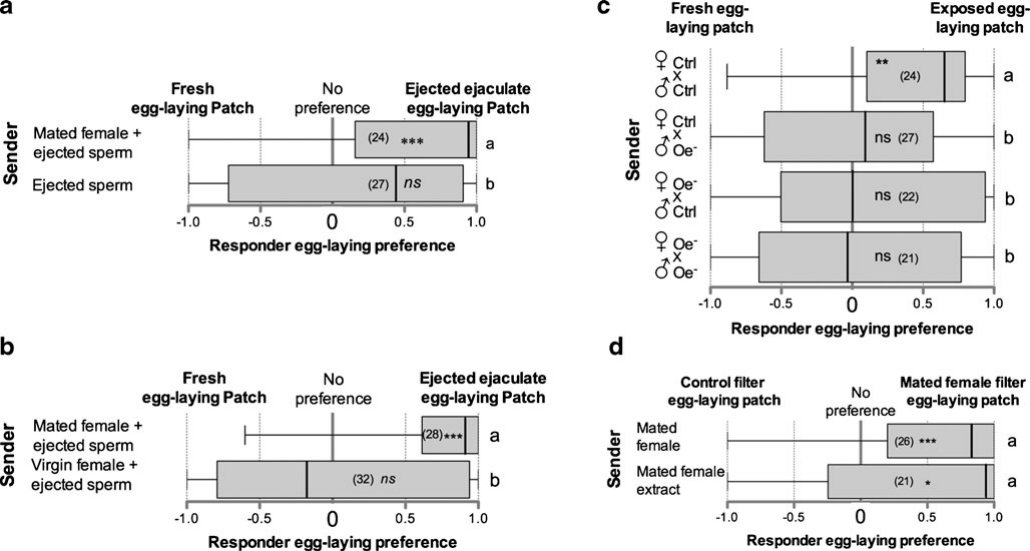
Ejected sperm and cuticular hydrocarbons attract egg laying. **a** Egg-laying preference of mated females between two food patches, one of which was previously exposed to a mated female or had received ejected sperm. To control for manipulation, the ejected sperm from the mated sender female was removed and replaced with that of another mated female. **b** Egg-laying patch preference of mated females between two food patches, one of which was previously exposed to a mated or virgin female. The food patch exposed to the virgin female received an ejected sperm from a mated female. **c** Control (Ctrl) females mated with Ctrl or oenocyte-less (Oe^−^) male, or Oe^−^ females mated with Ctrl or Oe^−^ males. **d** Egg-laying patch preference of mated female between two food patches, one of which had filter paper impregnated with the extract of one mated female, and the other a filter paper treated with solvent only. Preference for a site is indicated in each box plot by asterisks as determined by a two-tailed exact Wilcoxon signed rank test (ns: non-significant; ***, *P* < 0.001). Different letters to the right of the box plots indicate differences between groups, as determined by a logistic regression; see Table S4 for full statistics

To investigate the cues left by mated female senders, we tested whether ejected sperm was sufficient to indicate a favorable egg-laying patch. In a design similar to the one in Fig. 1a, we either forced a sender mated female to spend time and eject sperm on a high quality egg-laying patch or manually transferred freshly ejected sperm on a high-quality patch, and presented these marked patches to a responder female, each against an unmarked patch. While responder females preferentially laid eggs on patches previously occupied by a mated female and marked by sperm ejection, responders showed no preference to patches containing only ejected sperm (Fig. 3a). Ejection of sperm was, therefore, not sufficient to signal a favorable site. In a similar experiment, we forced a virgin female to occupy a high quality patch and supplemented this patch with ejected sperm from a mated female (Fig. 3b). Even though a female, albeit virgin, had occupied the patch, responder females still did not prefer that patch (Fig. 3b). Thus, responder females behaved differently when confronted with a potential egg-laying patch previously occupied by a virgin or by a mated female, regardless of the presence or absence of ejected sperm.

That cVA attracts flies to a food source (Bartelt et al. 1985; Lebreton et al. 2015), but ejected sperm containing cVA are not sufficient to indicate egg-laying sites (Fig. 3a, b), suggests that females signal favorable egg-laying sites using other chemical cues (in addition to cVA). We investigated CHC pheromones, which signal the sex and species of individual flies, in enabling sender mated females to guide responder females. We used male and female flies devoid of CHCs through ablation of their oenocytes. Oenocyte-less (Oe^−^) males produce cVA but lack CHCs (Billeter et al. 2009). Since females acquire additional CHCs together with cVA through mating (Fig. 2i; Table S2), we tested the contribution of both male and female CHCs by mating Oe^−^ and control (Ctrl) flies in a combinatorial design. Mated females were forced to occupy and eject male ejaculate on a high quality patch according to Fig. 1a. While Ctrl females mated with Ctrl males attracted responder females, neither Ctrl females mated with Oe^−^ males nor Oe^−^ females mated with Ctrl males influenced the egg-laying preferences of responder females (Fig. 3c). Analysis of ejected sperm from Oe^−^ females mated with Oe^−^ males showed an absence of CHCs but with wild-type levels of cVA (Table S1). These data indicate that social transmission of information about egg-laying sites relies on a blend of cVA supplied through the male sperm and both male and female CHCs.

Ejected sperm contains both cVA and CHCs (Fig. 2h), yet is not sufficient to attract responder females. Cuticular hydrocarbons must therefore be deposited by means other than sperm ejection. It is known that flies leave CHCs in the environment they occupy (Farine et al. 2012; Lin et al. 2015). Therefore, in order to determine whether mated females deposit CHCs, we housed mated or virgin females in a glass dish for 4 hr. Chemical analysis of the hexane extract of the dish revealed the presence of CHCs deposited by both mated and virgin females. Mated females deposited the male pheromone cVA and increased amounts of 7-T in addition to the female-specific pheromone components 7,11-HD and 7,11-ND, which also were deposited by virgin females (Table S3). To test the conclusion that mated sender females attract other flies purely through chemical communication, we extracted pheromones from a recently mated female that had not yet ejected, and thus contained both female pheromones and chemicals contained in the male sperm, and placed the extract on filter paper in a high quality patch and pitted this against another high quality patch marked with an identical paper impregnated with solvent only. The marked patch was preferred for egg laying by responder females over the control patch (Fig. 3d). Strikingly, the impregnated filter paper alone had a similar ability to attract a responder to a preferred egg-laying site as did a mated female, confirming that mated female senders share information with responders *via* chemical cues.

### Responder Females Use a Multisensory System to Select Egg-Laying Sites

**Fig. 4:**
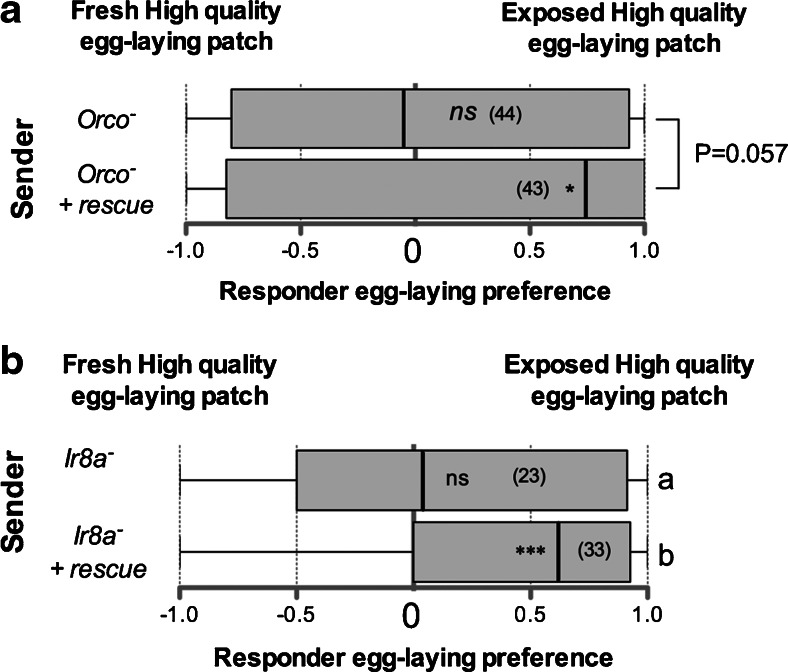
Responder females use multiple sensory systems to assess social cues. Egg-laying preferences of responder females between two patches, one of which was previously visited by a mated sender female. **a** Responder females are of the *Orco* ^−^ mutant genotype or “*Orco* ^−^ + *rescue* “in which a wild-type allele of *Orco* ^+^ was introduced. **b** Egg-laying site preferences of *Ir8a* ^−^ or Ir8a^−^ + *rescue* mated females between two food patches, one of which was visited by a mated *Oregon*-*R* female. Numbers of replicates are in brackets. Preference for a site is indicated in each box plot by asterisks (n.s.: not significant; 8 *, *P* < 0.05; ***, *P* < 0.001), as determined by a two-tailed exact Wilcoxon signed rank test. Different letters to the right of the box plots indicate differences between groups, as determined by a logistic regression; see Table S4 for full statistics

Having shown that mated females use a mixture of chemicals to influence the egg-laying choices of other mated females, we investigated the sensory channels used by responder females to sense cues left by sender mated females. We first tested responder females mutant for the *Orco* gene, which encodes a co-receptor essential for the function of classical odorant receptors (Larsson et al. 2004). *Orco*^−^ mutant females failed to respond to the cues left by sender mated females (Fig. 4a), while introduction of a wild-type copy of *Orco*^+^ in an *Orco*^−^ mutant genome rescued the response, indicating that females require an intact olfactory system for detecting social information about favorable egg-laying sites. This result is not surprising, given that several of the classical odorant receptors respond to both fly (van der Goes van Naters and Carlson 2007) and yeast odors (Christiaens et al. 2014; Stökl et al. 2010). *Orco* is, however, not required for the function of ionotropic receptors (Irs), a second family of odorant receptors (Benton et al. 2009). We tested females mutant for *Ir8a*, a gene encoding a co-receptor necessary for the function of half of the Irs, including those mediating the olfactory response to several identified yeast fermentation products (Silbering et al. 2011). *Ir8a*^−^ mutant females failed to respond to an egg-laying patch marked by a sender mated female (Fig. 4b), while introduction of a wild-type copy of *Ir8a*^+^ in an *Ir8a*^−^ mutant genome rescued the response, indicating that females require an intact ionotropic system to respond to senders. Given that Ir8a is involved in the sensing of several yeast-derived products, this suggests that responder females may evaluate the presence of yeast when deciding whether to follow a pheromonal mark. Our data indicate that responder females use at least two elements of their olfactory system to respond to sender females, which further indicates that social learning about egg-laying sites can occur *via* chemical cues.

## Discussion

We showed that mated female *D. melanogaster* can influence egg-laying by other females through marking food patches with a blend of male and female chemicals. Although we have not identified the exact combination of compounds that forms this egg-laying cue, we causally link CHCs made by both males and females with the bioassay response. *Drosophila* pheromones made in tissues other than oenocytes, such as cVA (Brieger and Butterworth 1970; Dweck et al. 2015; Shankar et al. 2015) are, therefore, not sufficient to attract egg-laying. This was further demonstrated by the lack of egg-laying preference induced by ejected sperm, which is laced with cVA, and the fact that mated females without oenocytes possess cVA but fail to influence oviposition by responder females. The lack of activity of cVA is, at first, puzzling given that this volatile pheromone has been linked previously with medium-range (5–20 m) attraction to food (Bartelt et al. 1985; Lebreton et al. 2012, 2015; Wertheim et al. 2002a). The lack of influence of cVA likely is due to the small size (90 mm diam.) of our oviposition arena. In the confine of this arena, CHCs, which are of limited volatility (Farine et al. 2012), are more informative as a close-range cue, and could function either as short-distance attractants, influencing orientation of the fly, or arrestants that result in a fly stopping upon contact (Kennedy 1978). Tracking responder flies on food patches exposed to mated females indicated that flies do not stop for long periods of times on a given food patch, suggesting that oviposition cues act as short-range attractants rather than arrestants. A recent study demonstrated that male *Drosophila* deposit (*Z*)-9-Tricosene (9-T) on substrates when sensing food odors, which attracts other flies and stimulates females to lay eggs on the substrate (Lin et al. 2015). This supports the idea that CHCs are part of the cue that acts as an egg-laying attractant. However, our findings indicate that mated females leave cues that are preferred over those left by males for guiding other mated females to an egg-laying site. This implies that additional CHC cues function in concert with 9-T to guide egg laying. This blend likely is a mixture of male- and female-specific CHCs. Mated females acquire the male part of the blend through male ejaculate and probably through contact during mating. The functional explanation for the higher response of females to mated females than to males may be linked to the greater reliability of egg-laying cues arising from mated females. This is because females can only obtain the male part of the blend through mating, thus guaranteeing their mating status, and because mated females have a clear preference for positioning themselves near high-quality egg-laying sites.

Chemical cues deposited by females act as a public source of information about egg-laying sites affecting the behavior of conspecific males and females: they attract both sexes and particularly guide mated females to lay eggs nearby. These cues might be provided inadvertently by individuals rubbing their cuticle (Everaerts et al. 2010; Levine et al. 2010), but also could be actively deposited thus conveying social information about egg-laying sites (Danchin et al. 2004; Symonds and Wertheim 2005). The costs and benefits of communal egg-laying mediated by these cues probably militate against active deposition. The oviposition cues benefit both senders and responders under low-density conditions by promoting communal egg-laying, resulting in higher offspring survival. However they are disadvantageous to both sender and responder females in high-density and low food conditions (Wertheim et al. 2002a, b). While signal reliability would be high if females were unable to control oviposition cue deposition and passively shed pheromones wherever they spend time, an ability to deposit pheromones actively would allow females to regulate group size in a density- and food quality-dependent manner. Interestingly, sperm ejection is under control of the brain (Lee et al. 2015), opening up the possibility that females control when, where and how much ejaculate they eject. A previous study indicated that aggregation in response to cVA changes, depending on substrate quality (Wertheim et al. 2002a), indicative of plasticity in either the response to sender or in the blend of pheromones deposited by the sender. The responder flies do not blindly respond to sender mated females, as demonstrated by the observation that responder females, given a choice between a poor egg-laying quality site marked by a sender mated female and an unexposed high quality egg-laying patch, lay eggs on both patches and not only on the marked patch. That responder females evaluate both egg-laying cues left by sender and food quality is indirectly suggested by the necessity of both the ionotropic odorant co-receptor Ir8a, involved in the sensing of several yeast-derived products (Silbering et al. 2011), and the classical odorant co-receptor Orco, required for the functioning of odorant receptors for fly odors (Lin et al. 2015; van der Goes van Naters and Carlson 2007). As Ir8a is necessary for females to adjust their sexual receptivity in response to the smell of yeast (Gorter et al. 2016), and classical odorant receptors are necessary for full receptivity in response to male courtship and, thus, probably male pheromones (Sakurai et al. 2013). We surmise that these two olfactory channels, respectively, assess the amount of yeast and the presence of fly pheromones in an egg-laying patch. Communication about egg-laying sites thus appears to be a balance between food patch quality and the transfer of social information mediated by chemical cues. Since the costs and benefits of communal egg-laying are density dependent (Wertheim et al. 2002b), a tight regulation of chemical cues deposited by senders and regulated response to these cues by a responder may have been favored by evolution.

## Electronic Supplementary Material

Below is the link to the electronic supplementary material.

ESM 1 (DOC 48 kb)

ESM 2 (DOC 44 kb)

ESM 3 (DOC 43 kb)

ESM 4 (DOC 107 kb)

ESM 5 (DOC 76 kb)

ESM 6 (DOC 66.4 kb)

## References

[CR1] Abuin L, Bargeton B, Ulbrich MH, Isacoff EY, Kellenberger S, Benton R (2011). Functional architecture of olfactory ionotropic glutamate receptors. Neuron.

[CR2] Bandura A (1971). Social learning theory.

[CR3] Bartelt RJ, Schaner AM, Jackson LL (1985). *Cis*-Vaccenyl acetate as an aggregation pheromone in Drosophila melanogaster. J Chem Ecol.

[CR4] Battesti M, Moreno C, Joly D, Mery F (2012). Spread of social information and dynamics of social transmission within Drosophila groups. Curr Biol.

[CR5] Baumberger JP (1917). The food of Drosophila melanogaster Meigen. Proc Natl Acad Sci USS.

[CR6] Becher PG, Flick G, Rozpędowska E, Schmidt A, Hagman A, Lebreton S, Larsson MC, Hansson BS, Piškur J, Witzgall P, Bengtsson M (2012). Yeast, not fruit volatiles mediate Drosophila melanogaster attraction, oviposition and development. Funct Ecol.

[CR7] Benton R, Vannice KS, Gomez-Diaz C, Vosshall LB (2009). Variant ionotropic glutamate receptors as chemosensory receptors in Drosophila. Cell.

[CR8] Billeter J-C, Levine JD (2013). Who is he and what is he to you? Recognition in Drosophila melanogaster. Curr Opin Neurobiol.

[CR9] Billeter J-C, Atallah J, Krupp JJ, Millar JG, Levine JD (2009). Specialized cells tag sexual and species identity in Drosophila melanogaster. Nature.

[CR10] Brieger G, Butterworth FM (1970). Drosophila melanogaster: identity of male lipid in reproductive system. Science.

[CR11] Butterworth FM (1969). Lipids of Drosophila: a newly detected lipid in the male. Science.

[CR12] Christiaens JF, Franco LM, Cools TL, De Meester L, Michiels J, Wenseleers T, Hassan BA, Yaksi E, Verstrepen KJ (2014). The fungal aroma gene ATF1 promotes dispersal of yeast cells through insect vectors. Cell Rep.

[CR13] Danchin É, Giraldeau L-A, Valone TJ, Wagner RH (2004). Public information: from nosy neighbors to cultural evolution. Science.

[CR14] Dweck HKM, Ebrahim SAM, Thoma M, Mohamed AA, Keesey IW, Trona F, Lavista-Llanos S, Svatoš A, Sachse S, Knaden M, Hansson BS (2015). Pheromones mediating copulation and attraction in Drosophila. Proc Natl Acad Sci U S A.

[CR15] Etienne R, Wertheim B, Hemerik L, Schneider P, Powell J (2002). The interaction between dispersal, the Allee effect and scramble competition affects population dynamics. Ecol Model.

[CR16] Everaerts C, Farine J-P, Cobb M, Ferveur J-F (2010). Drosophila cuticular hydrocarbons revisited: mating status alters cuticular profiles. PLoS ONE.

[CR17] Fan Y, Zurek L, Dykstra MJ, Schal C (2003). Hydrocarbon synthesis by enzymatically dissociated oenocytes of the abdominal integument of the German Cockroach, Blattella germanica. Naturwissenschaften.

[CR18] Farine J-P, Ferveur J-F, Everaerts C (2012). Volatile Drosophila cuticular pheromones are affected by social but not sexual experience. PLoS ONE.

[CR19] Gorter JA, Jagadeesh S, Gahr C, Boonekamp JJ, Levine JD, Billeter JC (2016). The nutritional and hedonic value of food modulate sexual receptivity in Drosophila melanogaster females. Sci Rep.

[CR20] Gou B, Liu Y, Guntur AR, Stern U, Yang CH (2014). Mechanosensitive neurons on the internal reproductive tract contribute to egg-laying-induced acetic acid attraction in Drosophila. Cell Rep.

[CR21] Jayaramaiah Raja S, Renkawitz-Pohl R (2005). Replacement by Drosophila melanogaster Protamines and Mst77F of histones during chromatin condensation in late spermatids and role of Sesame in the removal of these proteins from the male Pronucleus. Mol Cell Biol.

[CR22] Joseph RM, Devineni AV, King IFG, Heberlein U (2009). Oviposition preference for and positional avoidance of acetic acid provide a model for competing behavioral drives in Drosophila. Proc Natl Acad Sci U S A.

[CR23] Kennedy JS (1978). The concepts of olfactory ‘arrestment’ and “attraction”. Physiol Entomol.

[CR24] Krupp JJ, Kent C, Billeter J-C, Azanchi R, So AKC, Schonfeld JA, Smith BP, Lucas C, Levine JD (2008). Social experience modifies pheromone expression and mating behavior in male Drosophila melanogaster. Curr Biol.

[CR25] Laland KN (2004). Social learning strategies. Learn Behav.

[CR26] Larsson MC, Domingos AI, Jones WD, Chiappe ME, Amrein H, Vosshall LB (2004). Or83b Encodes a broadly expressed odorant receptor essential for Drosophila olfaction. Neuron.

[CR27] Laturney M, Billeter J-C (2014). Neurogenetics of female reproductive behaviors in Drosophila melanogaster. Adv Genet.

[CR28] Lebreton S, Becher PG, Hansson BS, Witzgall P (2012). Attraction of Drosophila melanogaster males to food-related and fly odours. J Insect Physiol.

[CR29] Lebreton S, Trona F, Borrero-Echeverry F, Bilz F, Grabe V, Becher PG, Carlsson MA, Nässel DR, Hansson BS, Sachse S, Witzgall P (2015). Feeding regulates sex pheromone attraction and courtship in Drosophila females. Sci Rep.

[CR30] Lee K-M, Daubnerová I, Isaac RE, Zhang C, Choi S, Chung J, Kim YJ (2015). A neuronal pathway that controls sperm ejection and storage in female Drosophila. Curr Biol.

[CR31] Levine J, Billeter J-C, Krull U, Sodhi R (2010). The cuticular surface of D. melanogaster: ToF-SIMS on the fly. Surf Interface Anal.

[CR32] Lin C-C, Prokop-Prigge KA, Preti G, Potter CJ (2015). Food odors trigger Drosophila males to deposit a pheromone that guides aggregation and female oviposition decisions. eLife.

[CR33] Lung O, Wolfner MF (2001). Identification and characterization of the major Drosophila melanogaster mating plug protein. Insect Biochem Mol Biol.

[CR34] Manier MK, Belote JM, Berben KS, Novikov D, Stuart WT, Pitnick S (2010). Resolving mechanisms of competitive fertilization success in Drosophila melanogaster. Science.

[CR35] Ribeiro C, Dickson BJ (2010). Sex peptide receptor and neuronal TOR/S6K signaling modulate nutrient balancing in Drosophila. Curr Biol.

[CR36] Sakurai A, Koganezawa M, Yasunaga K-I, Emoto K, Yamamoto D (2013). Select interneuron clusters determine female sexual receptivity in Drosophila. Nat Commun.

[CR37] Sarin S, Dukas R (2009). Social learning about egg-laying substrates in fruitflies. Proc Royal Soc B: Biol Sci.

[CR38] Schneider CA, Rasband WS, Eliceiri KW (2012). NIH Image to ImageJ: 25 years of image analysis. Nat Methods.

[CR39] Scott D (1986). Sexual mimicry regulates the attractiveness of mated Drosophila melanogaster females. Proc Natl Acad Sci U S A.

[CR40] Shankar S, Chua JY, Tan KJ, Calvert ME, Weng R, Ng WC, Mori K, Yew JY (2015). The neuropeptide tachykinin is essential for pheromone detection in a gustatory neural circuit. eLife.

[CR41] Silbering AF, Rytz R, Grosjean Y, Abuin L, Ramdya P, Jefferis GS, Benton R (2011). Complementary function and integrated wiring of the evolutionarily distinct Drosophila olfactory subsystems. J Neurosci.

[CR42] Stökl J, Strutz A, Dafni A, Svatos A, Doubsky J, Knaden M, Sachse S, Hansson BS, Stensmyr MC (2010). A deceptive pollination system targeting drosophilids through olfactory mimicry of yeast. Curr Biol.

[CR43] Symonds MRE, Wertheim B (2005). The mode of evolution of aggregation pheromones in Drosophila species. J Evol Biol.

[CR44] Terashima J, Bownes M (2004). Translating available food into the number of eggs laid by Drosophila melanogaster. Genetics.

[CR45] van der Goes van Naters W, Carlson JR (2007). Receptors and neurons for fly odors in Drosophila. Curr Biol.

[CR46] Wertheim B, Dicke M, Vet LEM (2002). Behavioural plasticity in support of a benefit for aggregation pheromone use in Drosophila melanogaster. Entomol Exp Appl.

[CR47] Wertheim B, Marchais J, Vet LEM, Dicke M (2002). Allee effect in larval resource exploitation in Drosophila: an interaction among density of adults, larvae, and micro-organisms. Ecol Entomol.

[CR48] Yang CH, Belawat P, Hafen E, Jan LY, Jan YN (2008). Drosophila egg-laying site selection as a system to study simple decision-making processes. Science.

