# Supplementary material for: Pheromonal Cues Deposited by Mated Females Convey Social Information about Egg-Laying Sites in Drosophila Melanogaster
Source: J Chem Ecol. 2016 Mar 19;42:259–69. doi: 10.1007/s10886-016-0681-3 (PMC4839039; doi:10.1007/s10886-016-0681-3)
Supplement: Supplementary file 1 — (DOC 48 kb) [file 10886_2016_681_MOESM1_ESM.doc]

|  | | **Compound** | **Oregon-R (15)** | | | | **Control (14)** | | | | | **Oenocyteless(14)** | | |
| --- | --- | --- | --- | --- | --- | --- | --- | --- | --- | --- | --- | --- | --- | --- |
| *1* | *c*VA | | 56,8 | ± | 11,0 |  | 65,90 | ± | 7,26 |  | 82,58 | | ± | 11,3 |
| 2 | 7-Tricosene | | 3,4 | ± | 0,7 |  | 2,52 | ± | 0,46 |  | 0 | | ± | 0 |
| 3 | nC23 | | 4,0 | ± | 0,7 |  |  |  |  |  |  | |  |  |
| 4 | 7-Pentacosene | | 3,9 | ± | 0,8 |  |  |  |  |  |  | |  |  |
| 5 | 7,11-HD | | 7,0 | ± | 1,2 |  | 3,0 | ± | 0,82 |  | 0 | | ± | 0 |
| 6 | 2-Me C26 | | 4,9 | ± | 0,8 |  | 0.88 | ± | 0,4 |  | 0 | | ± | 0 |
| 7 | 9-Heptacosene | | 1,0 | ± | 0,3 |  |  |  |  |  |  | |  |  |
| 8 | nC27 | | 2,0 | ± | 0,8 |  |  |  |  |  |  | |  |  |
| 9 | nC28 | | 1,2 | ± | 0,6 |  | 1,38 | ± | 0,32 |  | 0 | | ± | 0 |
| 10 | 7,11-ND | | 3,3 | ± | 0,7 |  |  |  |  |  |  | |  |  |
| 11 | 2-Me C28 | | 0,4 | ± | 0,1 |  |  |  |  |  |  | |  |  |
| 12 | 2-Me C30 | | 0,6 | ± | 0,3 |  |  |  |  |  |  | |  |  |

**Table S1**: **Chemical content of single ejected ejaculate.** Mean (ng) +/- SEM of compounds detected in sperm ejection extracts from Oregon-R females mated with Oregon-R males, Control females mated with Control males and Oenocyteless female mated with Oenocyteless males. Only compounds that could be reliably quantified are shown.
