# Supplementary material for: Pheromonal Cues Deposited by Mated Females Convey Social Information about Egg-Laying Sites in Drosophila Melanogaster
Source: J Chem Ecol. 2016 Mar 19;42:259–69. doi: 10.1007/s10886-016-0681-3 (PMC4839039; doi:10.1007/s10886-016-0681-3)
Supplement: Supplementary file 2 — (DOC 44 kb) [file 10886_2016_681_MOESM2_ESM.doc]

|  |  | Mean ± SEM (ng) | | | *d.f.* | *Test* | *P value* |
| --- | --- | --- | --- | --- | --- | --- | --- |
|  |  | Mated (27) | Ejected (28) | Virgin (29) |
| 1 | CvA | 135.81 ± 17.65 ^a^ | 31.73 ± 4.16 ^b^ | . | 2 | 68.35 | <0.001 |
| 2 | 7-Tricosene | 52.14 ± 3.29 ^a^ | 41.36 ± 2.19 ^a^ | 20.91 ± 0.83 ^b^ | 2 | 52.417 | <0.001 |
| 3 | nC23 | 78.83 ± 4.40 | 69.61 ± 3.76 | 69.99 ± 2.39 | 2 | 2.735 | 0.255 |
| 4 | nC24 | 18.95 ± 1.47 | 17.43 ± 1.11 | 20.17 ± 1.08 | 2 | 1.898 | 0.387 |
| 5 | 2MeC24 | 37.21 ± 2.49 | 34.82 ± 2.26 | 32.33 ± 1.31 | 2 | 1.518 | 0.468 |
| 6 | 9-Pentacosene | 40.31 ± 2.31 | 36.35 ± 2.22 | 34.71 ± 1.39 | 2 | 4.203 | 0.122 |
| 7 | 7-Pentacosene | 74.50 ± 5.00 ^a^ | 62.77 ± 3.36 ^a^ | 46.05 ± 1.93 ^b^ | 2 | 32.555 | <0.001 |
| 8 | nC25 | 59.70 ± 4.08 | 53.59 ± 3.28 | 51.61 ± 1.84 | 2 | 1.284 | 0.526 |
| 9 | 7.11-HD | 201.52 ± 13.13 | 183.60 ± 10.84 | 198.20 ± 9.55 | 2 | 0.558 | 0.757 |
| 10 | 2MeC26 | 116.90 ± 7.51 | 105.30 ± 10.04 | 96.04 ± 5.38 | 2 | 3.871 | 0.144 |
| 11 | nC27 | 21.07 ± 1.61 | 18.03 ± 1.38 | 18.52 ± 0.82 | 2 | 2.123 | 0.346 |
| 12 | 7,11-ND | 81.72 ± 3.72 | 77.41 ± 5.13 | 81.26 ± 3.98 | 2 | 0.866 | 0.649 |
| 13 | 2MeC28 | 23.45 ± 1.99 | 21.41 ± 1.68 | 21.41 ± 0.85 | 2 | 0.376 | 0.829 |

**Table S2**: **Cuticular hydrocarbon profiles of single *Drosophila melanogaster* females extracted with Hexane and analyzed by Gas Chromatography**. Extracted females were either Virgin, mated (before ejection) or ejected. *Kruskal–Wallis one-way* ANOVA values are shown. Means with different letters are significantly different, according to *Dunn’s* two-tailed pairwise comparison within each compound. The number of replicates is indicated between brackets next to each group.
