# Supplementary material for: Pheromonal Cues Deposited by Mated Females Convey Social Information about Egg-Laying Sites in Drosophila Melanogaster
Source: J Chem Ecol. 2016 Mar 19;42:259–69. doi: 10.1007/s10886-016-0681-3 (PMC4839039; doi:10.1007/s10886-016-0681-3)
Supplement: Supplementary file 3 — (DOC 43 kb) [file 10886_2016_681_MOESM3_ESM.doc]

|  | |  | ***Mean*** ± **SEM (ng)** | |  | | |
| --- | --- | --- | --- | --- | --- | --- | --- |
|  | |  | Mated female | Virgin female | *d.f.* | *Test* | *P value* |
| 1 | CvA | | 14.71 ± 2.47 | . | . | . | . |
| 2 | 7-T | | 3.92 ± 0.71 | 0.20 ± 0.20 | 1 | 13.322 | 0.003 |
| 3 | nC23 | | 4.29 ± 0.76 | 3.03 ± 0.77 | 1 | 1.354 | 0.248 |
| 4 | 9-Pentacosene | | 0.90 ± 0.45 | 0.57 ± 0.32 | 1 | 0.253 | 0.48 |
| 5 | 7-Pentacosene | | 5.93 ± 0.91 | 2.16 ± 0.74 | 1 | 7.229 | 0.219 |
| 6 | nC25 | | 1.29 ± 0.56 | 1.10 ± 0.51 | 1 | 0.07 | 0.52 |
| 7 | 7.11HD | | 13.99 ± 1.31 | 14.79 ± 1.85 | 1 | 0.124 (A) | 0.969 |
| 8 | 2MeC26 | | 8.11 ± 1.08 | 7.20 ± 0.93 | 1 | 0.414 (A) | 0.414 |
| 9 | nC27 | | 3.79 ± 1.53 | 3.72 ± 1.48 | 1 | 0.061 | 0.955 |
| 10 | 7,11ND | | 5.35 ± 0.72 | 5.95 ± 1.06 | 1 | 0.221 (A) | 0.876 |

**Table S3: Cuticular hydrocarbon collected from glass dishes visited by mated or virgin females and analyzed by Gas Chromatography**. The data represent average amounts (ng) of individual cuticular hydrocarbon class with standard error of mean (SEM). (A) F values from *ANOVA Wilk's lambda* with *Tukey Kramer* *post hoc* test. Otherwise, *Kruskal Walli*s with *Dunns* *post hoc* test values are shown.
