# Supplementary material for: Pheromonal Cues Deposited by Mated Females Convey Social Information about Egg-Laying Sites in Drosophila Melanogaster
Source: J Chem Ecol. 2016 Mar 19;42:259–69. doi: 10.1007/s10886-016-0681-3 (PMC4839039; doi:10.1007/s10886-016-0681-3)
Supplement: Supplementary file 4 — (DOC 107 kb) [file 10886_2016_681_MOESM4_ESM.doc]

| Explanatory variable | | Estimate | Standard Error | | *t* value | *P* value | Deviance , *d.f*. | |
| --- | --- | --- | --- | --- | --- | --- | --- | --- |
|  |  |  |  |  |  |  | Null model | Final model |
| *Independent variable: Number of eggs laid by responders on both food patches* | | | | | | | | |
| **Various sender with forced choice (Fig.1B)** | |  | |  |  |  |  |  |
| Mated female | *Intercept* | 1.1605 | | 0.3240 | 3.582 | <0.001 | 2945.7 , 205 | 2804.4 , 201 |
|  | No fly | -1.0743 | | 0.3673 | -2.925 | 0.004 |  |  |
|  | VF | -1.1198 | | 0.4259 | -2.629 | 0.009 |  |  |
|  | MM | -0.4526 | | 0.4198 | -1.078 | 0.282 |  |  |
|  | VF+MM | -0.6923 | | 0.4380 | -1.581 | 0.115 |  |  |
| **Choice between two marked patches (Fig.1C)** | | | | | | | | |
| MFvsMM | *Intercept* | 0.3269 | | 0.3116 | 1.049 | 0.298 | 1418.2 , 66 | 1361.2 , 64 |
|  | MFvsVF | 0.8042 | | 0.4684 | 1.717 | 0.091 |  |  |
|  | MFvsVM | 0.4491 | | 0.4442 | 1.011 | 0.316 |  |  |
| **Sender with choice (Fig.1E)** | |  | |  |  |  |  |  |
| Mated female | *Intercept* | 0.5102 | | 0.272 | 1.876 | 0.065 | 1426.1, 76 | 13335.0, 75 |
|  | Virgin female | -0.9841 | | 0.3911 | -2.516 | 0.040 |  |  |
| **Sender and food quality (Fig.1F)** | |  | |  |  |  |  |  |
| No fly | *Intercept* | -1.1836 | | 0.2293 | -5.160 | <0.001 | 3465.6 , 179 | 3047.0 , 177 |
|  | Fly | 0.9091 | | 0.2377 | 3.823 | <0.001 |  |  |
|  | Yeast | 0.0107 | | 0.0030 | 3.551 | <0.001 |  |  |
|  | Fly*Yeast | -0.0042 | | 0.0055 | -0.768 | 0.743 |  |  |
| Fly+Yeast 100 | *Intercept* | 0.7329 | | 0.3050 | 2.403 | 0.017 | 3465.6 , 179 | 3043.2 , 174 |
|  | Fly+Yeast 50 | -0.4468 | | 0.4122 | -1.084 | 0.279 |  |  |
|  | Fly+Yeast 0 | -0.9758 | | 0.4242 | -2.300 | 0.022 |  |  |
|  | No fly+Yeast 100 | -0.7481 | | 0.4270 | -1.752 | 0.081 |  |  |
|  | No fly+Yeast 50 | -1.4641 | | 0.4120 | -3.554 | <0.001 |  |  |
|  | No fly+Yeast 0 | -1.9140 | | 0.4383 | -4.367 | <0.001 |  |  |
| No fly+Yeast 0 | *Intercept* | -1.1810 | | 0.3147 | -3.753 | <0.001 | 3465.6 , 179 | 3043.2 , 174 |
|  | No fly+Yeast 50 | -0.4499 | | 0.4192 | 1.073 | 0.281 |  |  |
|  | No fly +Yeast 100 | 1.1659 | | 0.4339 | -2.687 | 0.008 |  |  |
|  | Fly + Yeast 50 | 1.4672 | | 0.4194 | 3.498 | <0.001 |  |  |
|  | Fly + Yeast 0 | 0.9382 | | 0.4312 | 2.176 | 0.031 |  |  |
| Fly+Yeast 50 | *Intercept* | 0.2862 | | 0.2772 | 1.032 | 0.303 | 3465.6 , 179 | 3043.2 , 174 |
|  | No fly+Yeast 50 | -1.0731 | | 0.3919 | -2.596 | 0.010 |  |  |
| **Ejected ejaculate alone (Fig. 3A)** | |  | |  |  |  |  |  |
| Mated female | *Intercept* | 1.4171 | | 0.426 | 3.325 | 0.002 | 902.26 , 50 | 826.71 , 49 |
|  | Ejected ejaculate | -1.1 | | 0.5138 | -2.141 | 0.037 |  |  |
| **Sender with ejaculate (Fig. 3B)** | | | | | | | | |
| Mated female | *Intercept* | 2.0842 | | 0.3983 | 5.232 | <0.001 | 943.43 , 59 | 736.52 , 58 |
|  | Virgin female | -1.8624 | | 0.471 | -3.954 | <0.001 |  |  |
| **Sender with various pheromone profiles (Fig. 3C)** | | | | | | | | |
| Cf*Cm | *Intercept* | 1.1122 | | 0.3212 | 3.463 | 0.001 | 1740.6 , 93 | 1621.2 , 90 |
|  | Cf*Oem | -0.9445 | | 0.4014 | -2.353 | 0.021 |  |  |
|  | Oef*Cm | -0.9064 | | 0.4131 | -2.194 | 0.031 |  |  |
|  | Oef*Oem | -1.0212 | | 0.4417 | -2.312 | 0.023 |  |  |
| **Sender pheromone extract (Fig. 3D)** | |  | |  |  |  |  |  |
| Mated female | *Intercept* | 1.0766 | | 0.3862 | 2.788 | 0.007 | 731.51 , 46 | 725.62 , 45 |
|  | Female extract | 0.3388 | | 0.5472 | 0.619 | 0.538 |  |  |
| **Responder genotype (Fig. 4A&B)** | | | | | | | | |
| Orco | *Intercept* | -0.0756 | | 0.2503 | -0.302 | 0.763 | 1515.9, 86 | 1464.5, 85 |
|  | Orco-R | 0.6987 | | 0.3631 | 1.924 | 0.058 |  |  |
| Ir8a- | *Intercept* | 0.0911 | | 0.2774 | 0.328 | 0.744 | 792.03 , 55 | 724.82 , 54 |
|  | Ir8a-R | 0.8930 | | 0.3697 | 2.415 | 0.019 |  |  |
| *Independent variable: number of positions* | | | |  |  |  |  |  |
| **Sender with forced choice: responder preference (Fig. 2A)** | | | | | | | | |
| Mated female | *Intercept* | 0.657 | | 0.2258 | 2.91 | 0.006 | 4289.9 , 39 | 3598.3 , 38 |
|  | Virgin female | -0.9577 | | 0.3338 | -2.869 | 0.007 |  |  |
| **Sender with choice: food preference (Fig. 2B)** | | | |  |  |  |  |  |
| Mated female | *Intercept* | 1.6548 | | 0.6522 | 2.538 | 0.016 | 3912.4 , 33 | 3388.0 , 32 |
|  | Virgin female | -1.7527 | | 0.8001 | -2.191 | 0.036 |  |  |
| **Responder preference (Fig. 2H)** | | | | | | | | |
| Mated female | *Intercept* | 0.657 | | 0.3195 | 2.056 | 0.044 | 10537.6 , 64 | 9723.3 , 62 |
|  | Male | 1.16 | | 0.5476 | 2.119 | 0.038 |  |  |
|  | Virgin female | 0.3579 | | 0.459 | 0.78 | 0.438 |  |  |

**Table S4. Summary of statistical tests to compare responses between types of marking**. Quasibinomial logistic regression was applied on the number of eggs laid or the number of positions on both food patches (independent variable) over the total number of eggs. Data were arranged as a matrix of 2 vectors: Number of successes (number of eggs/positions on the marked food patch) and number of failures (number of eggs/positions on the other food patch). Explanatory variables include the type of marking applied on one of the food patch (Marked food patch) and the type of responder used. Only significantly different groups are reported in this table. MF: mated female; VF: virgin female; VM: virgin male; MM: mated male; C: control; Oe: Oenocyteless.
