# Supplementary material for: Pheromonal Cues Deposited by Mated Females Convey Social Information about Egg-Laying Sites in Drosophila Melanogaster
Source: J Chem Ecol. 2016 Mar 19;42:259–69. doi: 10.1007/s10886-016-0681-3 (PMC4839039; doi:10.1007/s10886-016-0681-3)
Supplement: Supplementary file 5 — (DOC 76 kb) [file 10886_2016_681_MOESM5_ESM.doc]

| Responder | Sender | *N* | Mean | *d.f.* | *Test* | *P value* |
| --- | --- | --- | --- | --- | --- | --- |
| **Forced choice (Fig.1B)** | | | | | | |
| Mated Female | No fly | 32 | 20.59 ± 1.08 |  |  |  |
|  | Female + Male | 35 | 15.69 ± 1.57 |  |  |  |
|  | Mated female | 30 | 19.70 ± 2.48 |  |  |  |
|  | Virgin female | 31 | 19.03 ± 1.93 |  |  |  |
|  | Mated male | 34 | 21.06 ± 1.78 | 161 | *F*=1.478 | 0.211 |
| **double marking (Fig. 1C)** | | | | | | |
| Mated Female | VF vs MF | 24 | 34.50 ± 3.17 |  |  |  |
|  | VM vs MF | 22 | 39.23 ± 3.40 |  |  |  |
|  | MM vs MF | 21 | 37.62 ± 2.95 | 64 | *F*= 0.587 | 0.559 |
| **Choice experiment (Fig. 1E)** | | | | | | |
| Mated Female | Mated Female | 49 | 16.43 ± 1.37 |  |  |  |
|  | Virgin Female | 28 | 26.71 ± 2.77 | 75 | *t*= 3.715 | <0.001 |
| **Food quality and presence (Fig.1F)** | |  |  |  |  |  |
| Mated Female | Yeast100+female | 28 | 25.75 2.61 |  |  |  |
|  | Yeast50+female | 33 | 23.67 2.66 |  |  |  |
|  | Yeast0+female | 28 | 24.61 3.16 |  |  |  |
|  | Yeast100 | 32 | 20.59 1.08 |  |  |  |
|  | Yeast50 | 30 | 29.13 2.39 |  |  |  |
|  | Yeast0 | 31 | 26.65 2.34 | 181 | *t*=1.478 | 0.208 |
| **Ejaculate alone (Fig. 3A)** | | | | | | |
| Mated Female | Mated Female | 24 | 22.21 ± 2.59 |  |  |  |
|  | Sperm Ejection | 27 | 28.04 ± 3.04 | 49 | *t*= 1.440 | 0.156 |
| **Sender with ejaculate (Fig. 3B)** | | | | | | |
| Mated Female | Mated Female | 28 | 25.18 ± 1.54 |  |  |  |
|  | Virgin Female | 32 | 22.06 ± 1.84 | 58 | *t*= 1.280 | 0.205 |
| **Sender CHs extract (Fig. 3D)** | | | | | | |
| Mated Female | Mated Female | 26 | 25.00 ± 2.58 |  |  |  |
|  | MF Extract | 21 | 25.86 ± 1.89 | 45 | *t*=0.256 | 0.798 |
| **CHs profiles (Fig. 3C)** | | | | | | |
| Mated Female | CF(*CM) | 23 | 33.91 ± 2.51 |  |  |  |
|  | CF(*OeM) | 26 | 40.15 ± 3.10 |  |  |  |
|  | OeF(*CM) | 22 | 41.23 ± 3.68 |  |  |  |
|  | OeF(*OeM) | 21 | 31.43 ± 2.16 | 93 | *F*= 2.351 | 0.078 |
| **Responder (Fig. 4A&B)** | | | | | | |
| IR8a- | Mated Female | 22 | 26.73 ± 2.64 |  |  |  |
| IR8a-R |  | 32 | 29.88 ± 2.08 | 54 | *t*= 0.9986 | 0.322 |
| Orco |  | 44 | 19.84 ± 2.06 |  |  |  |
| Orco-R |  | 43 | 20.19 ± 1.82 | 85 | *t*= 0.1256 | 0.900 |
|  |  |  |  |  |  |  |

**Table S5. Mean +/- SEM number of eggs laid by responders in the different experiments.** Wild type Oregon-R flies were tested unless stated otherwise. MM: Mated Male; MF: Mated Female; VF: Virgin Female; VM: Virgin Male. C: Control flies; Oe: Oenocyteless flies.Different treatment were compared using *independent sample t-test* or *ANOVA Wilk’s Lambda* followed by *Tukey posthoc two-tailed* multiple comparison.
