# Supplementary material for: Pheromonal Cues Deposited by Mated Females Convey Social Information about Egg-Laying Sites in Drosophila Melanogaster
Source: J Chem Ecol. 2016 Mar 19;42:259–69. doi: 10.1007/s10886-016-0681-3 (PMC4839039; doi:10.1007/s10886-016-0681-3)
Supplement: Supplementary file 6 — (DOC 66.4 kb) [file 10886_2016_681_MOESM6_ESM.pdf]

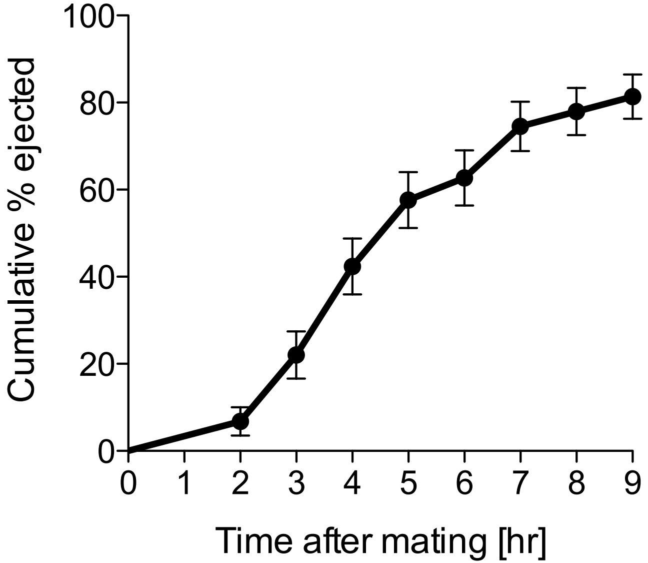

Figure S1. Timing of sperm ejection. Cumulative **hourly** percentage of *Oregon-R* females mated to Protamine-GFP males that ejected sperm after the end of mating (T=0). Number of replicate is 54. Error bars represent Standard error.
